# Receptor Tyrosine Kinases in Kidney Development

**DOI:** 10.1155/2011/869281

**Published:** 2011-03-03

**Authors:** Renfang Song, Samir S. El-Dahr, Ihor V. Yosypiv

**Affiliations:** Section of Pediatric Nephrology, Department of Pediatrics, Hypertension and Renal Center of Excellence, Tulane University Health Sciences Center, 1430 Tulane Avenue, New Orleans, LA 70112, USA

## Abstract

The kidney plays a fundamental role in the regulation of arterial blood pressure and fluid/electrolyte homeostasis. As congenital anomalies of the kidney and urinary tract (CAKUT) constitute one of the most common human birth defects, improved understanding of the cellular and molecular mechanisms that lead to CAKUT is critical. Accumulating evidence indicates that aberrant signaling *via* receptor tyrosine kinases (RTKs) is causally linked to CAKUT. Upon activation by their ligands, RTKs dimerize, undergo autophosphorylation on specific tyrosine residues, and interact with adaptor proteins to activate intracellular signal transduction pathways that regulate diverse cell behaviours such as cell proliferation, survival, and movement. Here, we review the current understanding of role of RTKs and their downstream signaling pathways in the pathogenesis of CAKUT.

## 1. Introduction

### 1.1. Brief Overview of Kidney Development

**Figure 1:**
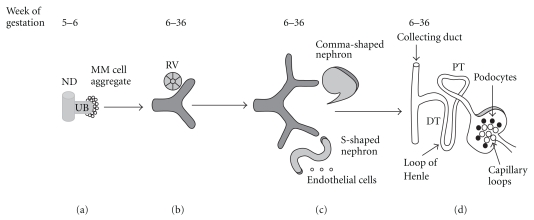
Schematic representation of normal development of the human kidney. (a) Invasion of the metanephric mesenchyme (MM) by the ureteric bud (UB) induces MM cells to aggregate around the UB tip. (b) MM cell aggregates undergo mesenchymal-to-epithelial transformation (MET) to form the renal vesicle (RV). (c) RV elongates along the proximal-distal axis to form comma-shaped and then S-shaped nephron. Distal ends of S-shaped nephrons fuse with UB-derived collecting ducts, whereas proximal clefts form glomeruli. Endothelial cells migrate into the proximal cleft. (d) Patterning of the S-shaped nephron and UB result in formation of mature nephron which contains glomerular capillary tuft, podocytes, proximal tubule (PT), loop of Henle, distal tubule (DT), and collecting duct. Please see text for details.

Development of the kidney and urinary tract begins when the nephric duct (ND) is formed from the intermediate mesoderm on embryonic (E) day E22 in humans and E8 in mice [6]. The ND extends caudally and forms an epithelial outgrowth called the ureteric bud (UB) (E28 in humans; E10.5 in mice), which invades the adjacent metanephric mesenchyme (MM) [6] (Figure 1). The permanent or metanephric kidney is formed *via* reciprocal inductive interactions between the UB and the MM [7, 8]. UB outgrowth from the ND is followed by its repetitive branching, a process called branching morphogenesis [9, 10]. Initial generations of UB branches will form the ureter, renal pelvis, and calyces, whereas subsequent generations of UB branches will give rise to collecting ducts. Distal ureter will translocate from the ND to eventually fuse with the bladder [11, 12]. Each UB tip is capable of inducing the adjacent metanephric mesenchyme to form nephrons (from the glomerulus through the distal tubule) [9]. UB signals to MM by secreting wingless 9b (Wnt9b), a soluble growth factor, which acts *via* canonical **β** catenin to induce expression of fibroblast growth factor 8 (FGF8), LIM homeobox 1 (Lhx1) and Wnt4 in the MM [7, 8, 13–15]. In turn, Wnt4 induces MM cells to undergo mesenchymal-to-epithelial transformation (MET) and differentiate into nephron epithelia [13].* Six2*, a homeodomain transcription factor expressed in the MM, maintains MM cells in undifferentiated state, thereby allowing continued UB branching and nephron formation to proceed [16]. Aberrant UB branching or nephron endowment is causally linked to congenital anomalies of the kidney and urinary tract (CAKUT), hypertension and eventual progression, to chronic renal failure [7, 17, 18]. Although some forms of CAKUT are a part of a syndrome, most cases of renal system anomalies are sporadic and isolated to the urinary tract.

### 1.2. Receptor Tyrosine Kinases (RTKs)

RTKs and their specific ligands are key regulators of critical cellular processes such as proliferation, survival, differentiation, and migration [19, 20]. Mutations in RTKs and aberrant activation of their signaling pathways have been causally linked to numerous diseases including cancers and CAKUT. For example, mutations rearranged during transfection (Ret) RTK in humans cause medullary thyroid carcinoma, breast cancer, and Hirschsprung disease [21, 22]. Humans have 58 known RTKs [23]. All RTKs have a common molecular architecture and are composed of ligand-binding domain in the extracellular region, a single transmembrane helix, a cytoplasmic region that contains the tyrosine kinase and C-terminal domains. Upon activation by their ligands, RTKs dimerize and undergo autophosphorylation on specific tyrosine residues leading to activation of intrinsic protein tyrosine kinase activity. In turn, these docking phosphotyrosines recruit specific intracellular adaptor proteins [phospholipase C gamma (PLC*γ*), growth factor receptor-bound protein 2 (Grb2), Src, Shc] and activate intracellular signal transduction pathways such as phosphatidylinositol 3-kinase (PI3K)/Akt, Ras/extracellular signal-regulated (Erk1/2) mitogen-activated protein (MAP) kinases or protein kinase C (PKC) [23, 24]. PI3Ks and MAP kinases are families of ubiquitously expressed proteins that mediate the effects of RTKs on cell proliferation, survival, differentiation, migration, and cytoskeletal reorganization [19, 20, 25]. In this review, we outline the role of RTKs and their respective ligands in normal and abnormal metanephric kidney development and discuss insights into the mechanisms by which aberrant RTK signaling causes CAKUT.

### 1.3. Genetic Mutations in RTKs Associated with CAKUT

**Figure 2:**
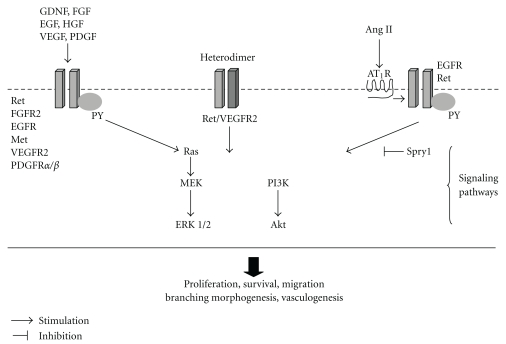
Schematic representation of the role of receptor tyrosine kinases (RTKs) in ureteric bud morphogenesis (UB) and kidney development. Growth factor ligands bind simultaneously with two cognate cell surface RTK molecules and cross-link them into a dimeric complex. This is followed by phosphorylation (P) of critical tyrosine (Y) residues of the intracellular tyrosine kinase domain, recruitment of specific docking proteins, and activation of intracellular signaling cascades. VEGFR2 heterodimerizes with Ret and induces P of ^Y1062^Ret [1]. Angiotensin (Ang) II, acting via the AT_1_ receptor (AT_1_R), transactivates EGFR and ^Y1062^Ret and stimulates their downstream signaling *via* the PI3K/Akt and Erk1/2 pathways to induce UB branching [2, 3]. In context of UB morphogenesis, induction of Ret activity is mediated by Ang II-induced downregulation of Spry1, a physiological inhibitor of RTK signaling [4, 5]. Please see text for details.

**Figure 3:**
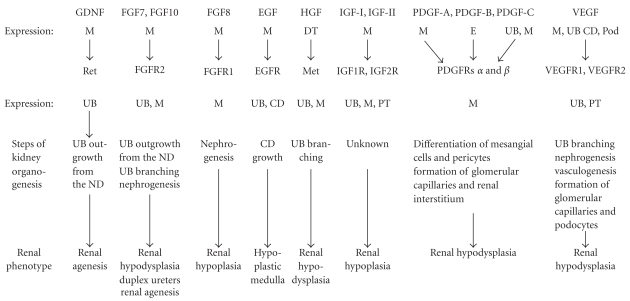
Schematic representation of the steps in kidney organogenesis requiring receptor tyrosine kinase signaling. M: mesenchyme, DT: distal tubule, E: vascular endothelial cells, UB: ureteric bud, CD: collecting duct, Pod: podocytes. Please see text for details.

Over the past decade, a number of RTKs and their respective ligands have been implicated in the control of metanephric kidney and urinary tract development. RTKs essential for normal metanephric development include rearranged during transfection (Ret), fibroblast growth factor receptor 2 (FGFR2), epidermal growth factor receptor (EGFR), hepatocyte growth factor (HGF) receptor, Met, vascular endothelial growth factor receptor 1 (VEGFR1/Flt1), vascular endothelial growth factor receptor 2 (VEGFR2/KDR), platelet-derived growth factor receptors *α* and *β* (PDGFR*α*, PDGFR*β*), and insulin growth factor (IGF) receptors (IGF1R, IGF2R) (Table 1, Figures 2 and 3). In addition to RTKs with known roles in kidney organogenesis, other RTKs with no specific renal functions assigned to them to date are also expressed in the developing kidney. For example, ROS1, the human homolog of the transforming gene v-ros of the avian sarcoma virus UR2, is expressed in distinctive fashion during kidney development in mice. On E11.0, ROS1 mRNA is present in the ND and the UB [46]. From E13.0 and throughout further development ROS1 is expressed specifically in UB tips. This spatial distribution suggests a direct role for ROS1 in inductive interactions between the UB and metanephric mesenchyme. Genetic inactivation of *ROS1* in mice leads to aberrant regionalization and terminal differentiation of epithelial cells in the epididymis, but does not appear to alter histology of the developing kidney [47]. Notably, ROS1-null male mice do not reproduce. This is presumably due to defects in sperm maturation or ability of sperm to fertilize *in vivo* [47]. RTK ligands that play an important role in kidney development include EGF, FGF7, FGF8, FGF10, GDNF, HGF, IGF1, PDGF-B, and VEGF (Table 2, Figure 3).

## 2. Ret

The glial-derived neurotrophic factor (GDNF)/Ret pathway is the major inducer of UB and kidney development [48]. GDNF, a growth factor secreted by metanephric mesenchymal cells, interacts with its RTK, Ret, a proto-oncogene expressed in the ND to induce epithelial cell rearrangement and migration leading to the formation of the primary UB [49]. In addition, GDNF stimulates Ret signaling in the UB tip cells to induce UB branching and nephron formation [50]. Genetic inactivation of GDNF or Ret in mice results in a failure to form a UB and perinatal death with bilateral kidney agenesis [26, 42]. Notably, mutations in Ret (OMIM#164762) are found in 35% of humans with various forms of renal agenesis [51]. Animal model systems provided valuable insights into the cellular and molecular mechanisms that regulate Ret. Ret expression and signaling activity is inhibited by Sprouty (Spry) 1 and induced by paired box 2 (Pax2) or retinoic acid (vitamin A) [4, 52, 53]. There are 2 isoforms of Ret RTK, Ret9, and Ret51. RET9 (1072 AAs) and Ret51 (1114 AAs) are identical in N-terminal domain and diverge after residue 1063 in the C-terminal cytoplasmic domain [54]. Ret activation results in phosphorylation (P) of key docking tyrosines (Y) that bind to several intracellular adaptor proteins such as Src (at Y981), PLC*γ* (at Y1015), SHC, Frs2, Irs1 and 2, ENIGMA, DOKs 4, 5 and 6 (at Y1062), and Grb2 (at Y1096) [55]. Recruitment of Src to Ret Y981 activates the MAPK pathway, while recruitment of PLC*γ* binding to Ret Y1015 induces the PKC pathway [55]. The Ret Y1062 phosphotyrosine serves as a docking site for Shc which recruits either the Grb2-Sos complex leading to MAPK activation or Grb2-GAB1 to stimulate PI3K/Akt signaling. Grb2 also binds to PY1096, where it preferentially activates PI3K/Akt signaling [56]. Recent elegant genetic studies in mice identified distinct roles of Ret RTK Y residues and their corresponding signaling pathways in the pathogenesis of diverse types of CAKUT [57]. Mutations in Y1015 of either Ret9 or Ret51 cause supernumerary ureters, renal hypodysplasia, and hydroureters. Interestingly, mutations in Y981 or Y1062 of Ret9 cause CAKUT (partial hydroureter and bilateral renal agenesis, resp.), whereas same mutations in Ret51 do not lead to CAKUT. Notably, mutations in Y1015 of Ret51 cause CAKUT alone, but mutations in Y981 or Y1062 of Ret9 result in CAKUT and Hirschsprung disease [58]. These findings indicate that signaling pathways downstream of Ret RTK have distinct and redundant roles in kidney development: PLC*γ* in ensuring only a single kidney is formed and PI3K/MAPK in promoting initiation of kidney formation. In addition, the ratio of Ret9/Ret51 isoforms may play an important role in the type and severity of CAKUT. 

What are the cellular mechanisms by which Ret signaling regulates UB and metanephric morphogenesis? Activation of Ret by GDNF induces cell proliferation in the UB tip cells [59, 60]. Localized cell proliferation may promote expansion of the UB tip and subsequent transformation of the rounded ampulla to a branched structure. Another mechanism may involve change in UB tip cell shape. Here, UB branching occurs through outpouching possibly mediated by wedge-shaped cells created through an apical cytoskeletal purse-string mechanism [61]. This possibility is further supported by the ability of actin depolymerizing factors, cofilin1 and destrin, to promote UB epithelial cells growth and branching [62]. Recent *in vivo* studies demonstrate that movement of Ret-expressing ND cells accounts for the UB outgrowth from the ND [49]. Induction of cell movement by Ret RTK is mediated by ETS transcription factors, Etv4 and Etv5 [63]. Although the mechanisms by which Ret signaling induces metanephric organogenesis remain to be fully elucidated, cell proliferation, migration, and shape change are a likely prerequisite for Ret-mediated UB branching and metanephric organogenesis.

## 3. Fibroblast Growth Factor Receptors

Fibroblast growth factor receptors (FGFRs) have four known signaling members and 22 ligands in mammals [64]. FGFRs are expressed throughout embryogenesis in many tissues, including the kidney and are critical in the development of multiple organs [64]. Since *FGFR1*- and FGFR2-null mice are early embryonic lethal [65, 66], conditional gene knockout approaches were utilized to reveal roles for these RTKs in kidney development. UB-specific inactivation of the FGFR2, which mediates signaling induced by FGF7 and FGF10, in mice reduces UB branching and nephron number [27, 67, 68]. These effects are mediated, in part, by FGFR2 substrate 2alpha (Frs2alpha), a major docking protein for FGFR2 [69]. Although *c-*Ret gene expression is not altered in FGFR2^UB-/-^ embryonic metanephroi [27], the full impact of FGFR2 deficiency on Ret signaling remains to be determined. Conditional inactivation of FGFR2 in the mesenchyme results in supernumerary UBs, duplex ureters, hydroureter, and renal agenesis [28]. Notably, mutations of FGFR2 in humans cause hydroureter and solitary kidney [70]. Interestingly, UB branching may be induced *in vitro* independently of Ret by the addition of FGF7 along with activin A, an activator of TGF-*β* signaling, or of FGF10 [49, 71]. Notably, FGF10 becomes critical for UB branching when Ret signaling is reduced or absent. In this regard, reduction in FGF10 gene dosage in double-transgenic GDNF^−/−^/Spry1^−/−^ mice results in renal agenesis [72]. GDNF^−/−^ or Ret^−/−^ mice exhibit bilateral kidney agenesis [26, 42], whereas Spry1^−/−^ mice develop supernumerary UB buds from the ND which subsequently develop into multiple ureters and kidneys [4]. Interestingly, double-transgenic GDNF^−/−^/Spry1^−/−^ or Ret^−/−^/Spry1^−/−^  mice form normal ureters and kidneys [72]. These findings indicate that in the absence of Spry1, a negative regulator of Ret signaling, Ret is no longer required for proper kidney development. Collectively, these observations suggest that when GDNF and Spry1 are both absent, FGF10/FGFR2 signaling allows UB branching and kidney development. Despite considerable similarities, renal phenotypes are not identical in FGF7- or FGF10-null compared to FGFR2^UB-/-^ mice. For example, UB branching defect is more severe in FGFR2^UB-/-^ than FGF7- or FGF10-null metanephroi [27, 38, 41]. In addition, FGFR2^UB-/-^ metanephroi exhibit defects in stromal patterning, a phenotype which was not reported in FGF7- or FGF10-null mice [27, 38, 41]. The phenotypic differences may be due to differences in background strains and therefore modifier genes. Another possibility is that collaborative effect of FGF7 and FGF10 (or FGFR2 ligands other than FGF7/FGF10) may be required for kidney morphogenesis. 

FGFRs activate several signaling pathways common to Ret [73]. For example, treatment of kidneys with exogenous FGF10, but not FGF7, induces Etv4/Etv5 expression [74]. Induction of Etv4/Etv5 in the UB is abrogated by inhibition of PI3K pathway (activated by both Ret and FGFRs) [74]. Since Etv4/Etv5 expression is not altered in FGFR2-null kidneys, they may be regulated primarily by Ret, but not FGFR2, signaling. However, the ability of Etv4^−/−^/Etv5^−/−^ ND cells to contribute to the UB in chimeric mouse kidneys is severely compromised compared with Ret*^−/−^* cells [63]. These observations suggest that Etv4/Etv5 not only function downstream of Ret in kidney development, but also have a broader role downstream of multiple RTKs.

## 4. Epidermal Growth Factor Receptor

Signaling through epidermal growth factor receptor (EGFR) is critical for fundamental cellular functions such as proliferation, migration, growth, and differentiation [75]. In addition to EGF, EGF-like growth factors (HB-EGF, amphiregulin, betacellulin, epigen, and epiregulin) bind to EGFR [76]. During metanephric development, EGFR is expressed in renal collecting ducts [29, 77]. Stimulation of EGFR with EGF enhances branching morphogenesis in murine inner medullary collecting duct (IMCD3) cells *in vitro* [36, 37]. Moreover, EGFR-mutant mice die within 1 week of postnatal life and exhibit abnormal collecting ducts [78]. Targeted inactivation of the EGFR in mice results in hypoplastic renal medulla, dilated collecting ducts, and reduced ability to concentrate urine [29]. These changes are accompanied by increased apoptosis of papillary epithelia. Collectively, these findings indicate that FGFR is essential for collecting duct growth/elongation and maturation of renal urine-concentrating ability. The signaling events linking EGFR activation and UB/collecting duct morphogenesis may include MAP kinase and PI3K pathways. In this regard, pharmacologic inhibition of Erk1/2 or PI3K attenuates EGF-induced renal epithelial morphogenesis *in vitro* [36].

## 5. Met

In the developing metanephros, hepatocyte growth factor (HGF) is expressed in the mesenchyme, whereas Met is localized in the mesenchyme and the UB [79]. Genetic inactivation of HGF or Met in mice results in early embryonic lethality due to liver and placental anomalies [80]. Targeted inactivation of Met in the UB causes decreased UB branching and reduced nephron number [30]. Interestingly, EGFR expression is induced in kidneys of Met^−/−^ compared with wild-type mice. This is accompanied by increased phosphorylation of Y992 and Y1068 of EGFR. Moreover, defects in UB branching and nephron number are more severe in double-mutant mice that lack both Met and *EGFR* compared with Met^−/−^ mice [30]. These findings indicate that Met and EGFR play complementary roles in collecting duct growth and nephrogenesis. In addition, Met and *α*3*β*1 integrin, a major laminin receptor essential for kidney development, signal coordinately to regulate the expression of Wnt7b which acts to promote normal renal papillary development [81]. Met signals *via* Erk1/2, PI3K, Pkc, Plc, Src, Fac, and Jak/Stat [82–85]. With respect to kidney development, activation of Erk1/2, PI3K, and PLC*γ* signaling pathways is critical for HGF-induced cell motility and branching process formation in IMCD-3 cells *in vitro* [36].

## 6. Vascular Endothelial Growth Factor Receptor 2

Vascular endothelial growth factor (VEGF) acts *via* VEGFR1 (Flt1) and VEGFR2 (KDR) to regulate endothelial cell proliferation, migration, and differentiation [86]. Most actions of VEGF are mediated *via* VEGFR2, whereas VEGFR1 is considered a “decoy” RTK. In the adult rat kidney, VEGFR2 is present in glomerular endothelial, distal tubule and collecting duct cells [87]. VEGF mRNA is expressed in both the UB and the mesenchyme in the developing mouse metanephros [31]. It is believed that VEGF released by the podocytes induces migration of vascular endothelial cells into the vascular cleft [32]. In turn, endothelial cells promote differentiation of renal mesangial cells *via* production of platelet-derived growth factor (PDGF) [88]. Exogenous VEGF stimulates nephrogenesis, UB branching and vasculogenesis [31, 32]. Notably, the effect of VEGF on UB morphogenesis is mediated *via* VEGFR2 [33]. Moreover, VEGFR2 physically interacts with Ret, and exogenous VEGF induces phosphorylation of Y1062 in Ret in UB-derived cells *in vitro* [1]. These findings suggest that cooperation of VEGFR2 and Ret may play an important role in renal collecting system development.

## 7. Platelet-Derived Growth Factor Receptors

Two PDGF receptors, PDGFR*α* and PDGFR*β*, mediate the effect of five PDGFs each functioning in dimeric configurations AA, BB, AB, CC, and DD [23, 89]. In the human fetal kidney, PDGF-C localized to the mesangium, UB and the mesenchyme [90]. In the adult kidney, PDGF-C is expressed in parietal epithelial cells of Bowman's capsule, loops of Henle, distal tubules, collecting ducts and in arterial endothelial cells [90]. Genetic inactivation of the *PDGFR*β** in mice results in lack of mesangial cells and pericytes of the glomerular capillaries [35]. Conditional inactivation of the *PDGFR*β** in mice causes podocyte hypertrophy, glomerular capillary collapse, and prevents age-related mesangial expansion [91]. *PDGFR*α**-null mice also exhibit decreased mesangial cell recruitment and defective stromal mesenchyme [34]. Therefore, one role of PDGF released by the glomerular endothelial cells during metanephrogenesis is to promote differentiation of mesangial cells and promote glomerular vasculogenesis [88].

## 8. Insulin Growth Factor Receptors

The insulin-like growth factor (IGF) family includes ligands (IGF1, IGF2, and insulin) and their respective receptors (IGF1R, IGF2R, and InsR). IGFs/IGFRs play an essential role in the regulation of cell growth, proliferation, survival and affect function of many organ systems. In the adult canine kidney, both IGF1R and IGF2R are detected in the proximal tubular cells by binding autoradiography [92]. IGF1R, IGF2R and insulin receptor (INSR) mRNA and protein are expressed ubiquitously in the developing metanephros as early as on embryonic (E) day 14.0 in the rat [93]. At later stages of gestation, IGF1R expression is enhanced in the proximal tubules. IGF1/2 mRNA is also present in the rat kidney from E13.0 and treatment of metanephroi with anti-IGF1/2 antibodies retards kidney growth *ex vivo* [94]. Interestingly, intrauterine growth restriction (IUGR) in rats is accompanied by a decreased glomerular number and reduced IGF2R protein expression [95]. In contrast, fetal kidney IGF1 and insulin protein levels are increased compared with nonmanipulated embryos. Therefore, IGFRs promote nephrogenesis and renal organogenesis, and may be causally linked to occurrence of renal hypodysplasia.

## 9. Renin-Angiotensin System-RTK Crosstalk in Kidney Development

Crosstalk with RTKs has been shown to mediate the growth factor-like effects of a number of G protein-coupled receptor (GPCR-) activating ligands, such as angiotensin (Ang) II. We recently examined the role of Ret and EGFR in Ang II-induced UB branching. The critical role of the renin-angiotensin system (RAS) in metanephric development is evident from the observations that genetic inactivation of its components, such as angiotensinogen (AGT), renin, angiotensin-converting enzyme (ACE), or Ang II receptors (AT_1_R, AT_2_R) in mice causes hydronephrosis, hypoplastic medulla, and papilla [96]. AT_2_R-mutant mice demonstrate increased incidence of duplex ureters and vesicoureteral reflux [97]. Mutations in the genes encoding for AGT, renin, ACE or AT_1_R in humans are associated with renal tubular dysgenesis (RTD) [98]. In RTD, collapsed collecting ducts and abundant interstitial fibrosis in the medulla are observed. Given that EGFR- and ^Y1062^Ret-mutant mice exhibit CAKUT, we recently examined the role of EGFR and ^Y1062^Ret in Ang-II-induced UB branching morphogenesis. Ang-II increases phosphorylation of Y1173 and Y845 of EGFR in UB-derived cells *in vitro* and of ^Y1062^Ret in whole embryonic metanephroi grown *ex vivo* [2, 3]. Notably, Y1173 and Y845 of EGFR are the major sites of EGFR autophosphorylation which occurs as a result of EGF binding [98]. Moreover, inhibition of EGFR activity with the specific antagonist AG1478 abrogates Ang-II-induced UB branching in the intact metanephros [2]. Since, ^Y1062^Ret serves as a docking site for protein complexes that activate PI3K/Akt and Erk1/2 signaling pathways [57, 99], we recently examined the ability of Ang II to induce phosphorylation of Akt. Treatment of whole intact E12.5 metanephroi or UB cells with Ang II increases phosphorylation of Akt [3]. Moreover, Ang-II-induced increase in UB branching is abrogated by pretreatment with the specific PI3K inhibitor LY294002 or Erk1/2 inhibitor PD98059 [3]. These findings indicate that PI3K/Akt and Erk1/2 are essential for Ang-II-induced UB branching. Since exogenous Ang II decreases the expression of Spry1 in whole embryonic mouse metanephroi and in UB cells cultured *in vitro*, the stimulatory effects of Ang II on EGFR and Ret Y phosphorylation and UB branching may be mediated *via *downregulation of Spry1, a physiological inhibitor of RTK signaling [5]. Collectively, our data indicate that stimulation of Ang II GPCRs by their ligand induces tyrosine phosphorylation of EGFR and Ret leading to enhanced UB morphogenetic signaling and branching. Thus, cooperation of Ang II receptor and EGFR/Ret signaling is important in the development of the renal collecting sustem.

## 10. Conclusions and Perspectives

RTKs are emerging as critical players in normal metanephric kidney development. It has become evident that not only the classical direct stimulation of RTKs by their cognate ligands, but also ligand-independent, through transactivation of RTKs by G protein-coupled receptors, or heterodimerization with other RTKs play an important role in UB branching and metanephric organogenesis. Even though significant progress have been made in defining the role of RTKs and specific contribution of RTK-dependent signal transduction in metanephric development, further characterization of the subcellular processes involved in RTK activation and signaling is needed. 

What are the major open questions regarding RTK signaling during kidney organogenesis? These include elucidation of how activation of RTK signaling affects the phosphoproteome in the developing kidney and the cell type-specific responses to signals transmitted by distinct signaling pathways activated by RTKs. What are the perspectives for systems-wide approaches to understand RTK signaling in kidney organogenesis? In this regard, application of new genetic tools, such as conditional/tissue/cell-specific gene knockouts, genetic lineage tracing, and fluorescent *in vivo* reporters of cell signaling, whole genome-wide analysis of gene regulatory networks (including epigenetic regulation) that control different aspects of kidney development (e.g., microarray, ChIP-Seq) should provide important insights in understanding molecular mechanisms that direct normal and abnormal metanephric kidney development. Defining molecular aberrations leading to CAKUT in animals and humans with mutations in genes encoding for RTKs and their cognate ligands will uncover biomarkers that can be used for early diagnosis or prevention of renal system anomalies in children. Finally, establishment of shared large biorepositories of patients encompassing a wide spectrum of CAKUT phenotypes for molecular, genetic, and translational studies will define clinically relevant mutations in RTKs, their ligands, and interacting genes. This information can then be used to develop new effective preventive or therapeutic interventions for CAKUT.

## Figures and Tables

**Table 1:** Receptor tyrosine kinases associated with aberrant kidney development.

RTK type	Renal phenotype	References
Ret	Renal agenesis	[26]
FGFR2^UB-/-^	Reduced UB branching and nephron number Defects in stromal patterning	[27]
FGFR2^Mes-/-^	Supernumerary UBs, duplex ureters, hydroureter renal agenesis	[28]
EGFR	Hypoplastic renal medulla, dilated collecting ducts reduced ability to concentrate urine	[29]
Met^UB-/-^	Reduced UB branching and nephron number	[30]
VEGFR2	Stimulates UB branching, nephrogenesis and vasculogenesis	[31–33]
PDGFR*α*	Decreased number of mesangial cell defective stromal mesenchyme	[34]
PDGFR*β*	Lack of mesangial cells and pericytes	[35]

RTK: receptor tyrosine kinase, Ret: rearranged during transfection, FGF: fibroblast growth factor, UB: ureteric bud, Mes: mesenchyme, EGF: epidermal growth factor, Met: hepatocyte growth factor receptor, VEGF: vascular endothelial growth factor, PDGF: platelet-derived growth factor.

**Table 2:** Ligands of receptor tyrosine kinases with demonstrated functions in kidney organogenesis.

Ligand	Model	Renal phenotype	References
EGF	IMCD3 cells	Induction of cell migration and process formation *in vitro *	[36]
	UB cells	Induction of branching morphogenesis *in vitro *	[37]
FGF7	FGF7^−/−^	Small kidneys, reduced UB branching and nephron number	[38]
FGF8	Pax3Cre/*FGF * ^loxP/loxP^	Small kidneys, reduced number of collecting duct tips lack of progression of nephrogenesis to the S-shaped body stage short tubules	[39]
	BrachyuryCre/*FGF * ^loxP/loxP^	Small kidneys, reduced UB branching, lack of progression of nephrogenesis to the comma- and S-shaped body stages	[40]
FGF10	FGF10^−/−^	Small kidneys, dysplasia of outer medulla	[41]
GDNF	GDNF^−/−^	Renal agenesis	[42]
HGF	IMCD3 cells	Induction of cell process formation *in vitro *	[36]
IGF1	UB cells	Induction of cell process formation *in vitro *	[43]
PDGF-B	PDGF-B^−/−^	Lack of mesangial cells and pericytes	[44]
VEGF	Rat metanephric organ culture	Induction of vasculogenesis and tubulogenesis *in vitro *	[32]
	Mouse metanephric organ culture	Induction of UB branching and nephrons	[31]
	Rat metanephric and isolated intact UB culture	Induction of UB branching	[33]
	NephrinCre/VEGF-A^loxP/loxP^	Small glomeruli, paucity of glomerular capillary loops effacement of podocyte foot processes	[45]

UB: ureteric bud, IMCD3: murine inner medullary collecting duct cells.

## References

[1] Tufro A, Teichman J, Banu N, Villegas G (2007). Crosstalk between VEGF-A/VEGFR2 and GDNF/RET signaling pathways. *Biochemical and Biophysical Research Communications*.

[2] Yosypiv IV, Schroeder M, El-Dahr SS (2006). Angiotensin II type 1 receptor-EGF receptor cross-talk regulates ureteric bud branching morphogenesis. *Journal of the American Society of Nephrology*.

[3] Song R, Spera M, Garrett C, Yosypiv IV (2010). Angiotensin II-induced activation of c-Ret signaling is critical in ureteric bud branching morphogenesis. *Mechanisms of Development*.

[4] Basson MA, Watson-Johnson J, Shakya R (2006). Branching morphogenesis of the ureteric epithelium during kidney development is coordinated by the opposing functions of GDNF and Sprouty1. *Developmental Biology*.

[5] Yosypiv IV, Boh MK, Spera MA, El-Dahr SS (2008). Downregulation of Spry-1, an inhibitor of GDNF/Ret, causes angiotensin II-induced ureteric bud branching. *Kidney International*.

[6] Saxen L (1987). *Organogenesis of the Kidney*.

[7] Schedl A (2007). Renal abnormalities and their developmental origin. *Nature Reviews Genetics*.

[8] Costantini F, Kopan R (2010). Patterning a complex organ: branching morphogenesis and nephron segmentation in kidney development. *Developmental Cell*.

[9] Ekblom P (1989). Developmentally regulated conversion of mesenchyme to epithelium. *FASEB Journal*.

[10] Grobstein C (1953). Inductive epithelio-mesenchymal interaction in cultured organ rudiments of the mouse. *Science*.

[11] Batourina E, Choi C, Paragas N (2002). Distal ureter morphogenesis depends on epithelial cell remodeling mediated by vitamin A and Ret. *Nature Genetics*.

[12] Batourina E, Tsai S, Lambert S (2005). Apoptosis induced by vitamin A signaling is crucial for connecting the ureters to the bladder. *Nature Genetics*.

[13] Stark K, Vainio S, Vassileva G, McMahon AP (1994). Epithelial transformation metanephric mesenchyme in the developing kidney regulated by Wnt-4. *Nature*.

[14] Carroll TJ, Park JS, Hayashi S, Majumdar A, McMahon AP (2005). Wnt9b plays a central role in the regulation of mesenchymal to epithelial transitions underlying organogenesis of the mammalian urogenital system. *Developmental Cell*.

[15] Iglesias DM, Hueber PA, Chu L (2007). Canonical WNT signaling during kidney development. *American Journal of Physiology*.

[16] Kobayashi A, Valerius MT, Mugford JW (2008). Six2 defines and regulates a multipotent self-renewing nephron progenitor population throughout mammalian kidney development. *Cell Stem Cell*.

[17] Brenner BM, Garcia DL, Anderson S (1988). Glomeruli and blood pressure. Less of one, more the other?. *American Journal of Hypertension*.

[18] Lisle SJM, Lewis RM, Petry CJ, Ozanne SE, Hales CN, Forhead AJ (2003). Effect of maternal iron restriction during pregnancy on renal morphology in the adult rat offspring. *British Journal of Nutrition*.

[19] Schlessinger J (2000). Cell signaling by receptor tyrosine kinases. *Cell*.

[20] Blume-Jensen P, Hunter T (2001). Oncogenic kinase signalling. *Nature*.

[21] Boulay A, Breuleux M, Stephan C (2008). The ret receptor tyrosine kinase pathway functionally interacts with the ER*α* pathway in breast cancer. *Cancer Research*.

[22] Iwashita T, Kurokawa K, Qiao S (2001). Functional analysis of RET with Hirschsprung mutations affecting its kinase domain. *Gastroenterology*.

[23] Lemmon MA, Schlessinger J (2010). Cell signaling by receptor tyrosine kinases. *Cell*.

[24] Madhani HD (2001). Accounting for specificity in receptor tyrosine kinase signaling. *Cell*.

[25] Cantley LG, Cantly LC (1995). Signal transduction by the hepatocyte growth factor receptor, c-met: activation of the phosphatidylinositol 3-kinase. *Journal of the American Society of Nephrology*.

[26] Schuchardt A, D’Agati V, Larsson-Blornberg L, Costantini F, Pachnis V (1994). Defects in the kidney and enteric nervous system of mice lacking the tyrosine kinase receptor Ret. *Nature*.

[27] Zhao H, Kegg H, Grady S (2004). Role of fibroblast growth factor receptors 1 and 2 in the ureteric bud. *Developmental Biology*.

[28] Hains D, Sims-Lucas S, Kish K, Saha M, Mchugh K, Bates CM (2008). Role of fibroblast growth factor receptor 2 in kidney mesenchyme. *Pediatric Research*.

[29] Zhang Z, Pascuet E, Hueber PA (2010). Targeted inactivation of EGF receptor inhibits renal collecting duct development and function. *Journal of the American Society of Nephrology*.

[30] Ishibe S, Karihaloo A, Ma H (2009). Met and the epidermal growth factor receptor act cooperatively to regulate final nephron number and maintain collecting duct morphology. *Development*.

[31] Gao X, Chen X, Taglienti M, Rumballe B, Little MH, Kreidberg JA (2005). Angioblast-mesenchyme induction of early kidney development is mediated by Wt1 and Vegfa. *Development*.

[32] Tufro A, Norwood VF, Carey RM, Gomez RA (1999). Vascular endothelial growth factor induces nephrogenesis and vasculogenesis. *Journal of the American Society of Nephrology*.

[33] Marlier A, Schmidt-Ott KM, Gallagher AR, Barasch J, Karihaloo A (2009). Vegf as an epithelial cell morphogen modulates branching morphogenesis of embryonic kidney by directly acting on the ureteric bud. *Mechanisms of Development*.

[34] Li X, Pontén A, Aase K (2000). PDGF-C is a new protease-activated ligand for the PDGF *α*-receptor. *Nature Cell Biology*.

[35] Soriano P (1994). Abnormal kidney development and hematological disorders in PDGF *β*- receptor mutant mice. *Genes and Development*.

[36] Karihaloo A, O’Rourke DA, Nickel C, Spokes K, Cantley LG (2001). Differential MAPK pathways utilized for HGF- and EGF-dependent renal epithelial morphogenesis. *Journal of Biological Chemistry*.

[37] Sakurai H, Tsukamoto T, Kjelsberg CA, Cantley LG, Nigam SK (1997). EGF receptor ligands are a large fraction of in vitro branching morphogens secreted by embryonic kidney. *American Journal of Physiology*.

[38] Qiao J, Uzzo R, Obara-Ishihara T, Degenstein L, Fuchs E, Herzlinger D (1999). FGF-7 modulates ureteric bud growth and nephron number in the developing kidney. *Development*.

[39] Grieshammer U, Cebrián C, Ilagan R, Meyers E, Herzlinger D, Martin GR (2005). FGF8 is required for cell survival at distinct stages of nephrogenesis and for regulation of gene expression in nascent nephrons. *Development*.

[40] Perantoni AO, Timofeeva O, Naillat F (2005). Inactivation of FGF8 in early mesoderm reveals an essential role in kidney development. *Development*.

[41] Ohuchi H, Hori Y, Yamasaki M (2000). FGF10 acts as a major ligand for FGF receptor 2 IIIb in mouse multi-organ development. *Biochemical and Biophysical Research Communications*.

[42] Sanchez MP, Silos-Santiago I, Frisén J, He B, Lira SA, Barbacid M (1996). Renal agenesis and the absence of enteric neurons in mice lacking GDNF. *Nature*.

[43] Sakurai H, Barros EJ, Tsukamoto T, Barasch J, Nigam SK (1997). An in vitro tubulogenesis system using cell lines derived from the embryonic kidney shows dependence on multiple soluble growth factors. *Proceedings of the National Academy of Sciences of the United States of America*.

[44] Levéen P, Pekny M, Gebre-Medhin S, Swolin B, Larsson E, Betsholtz C (1994). Mice deficient for PDGF B show renal, cardiovascular, and hematological abnormalities. *Genes and Development*.

[45] Eremina V, Sood M, Haigh J (2003). Glomerular-specific alterations of VEGF-A expression lead to distinct congenital and acquired renal diseases. *Journal of Clinical Investigation*.

[46] Sonnenberg E, Godecke A, Walter B, Bladt F, Birchmeier C (1991). Transient and locally restricted expression of the ros1 protooncogene during mouse development. *EMBO Journal*.

[47] Sonnenberg-Riethmacher E, Walter B, Riethmacher D, Gödecke S, Birchmeier C (1996). The c-ros tyrosine kinase receptor controls regionalization and differentiation of epithelial cells in the epididymis. *Genes and Development*.

[48] Brophy PD, Ostrom L, Lang KM, Dressler GR (2001). Regulation of ureteric bud outgrowth by Pax2-dependent activation of the glial derived neurotrophic factor gene. *Development*.

[49] Chi X, Michos O, Shakya R (2009). Ret-dependent cell rearrangements in the Wolffian duct epithelium initiate ureteric bud morphogenesis. *Developmental Cell*.

[50] Pachnis V, Mankoo B, Costantini F (1993). Expression of the c-ret proto-oncogene during mouse embryogenesis. *Development*.

[51] Skinner MA, Safford SD, Reeves JG, Jackson ME, Freemerman AJ (2008). Renal aplasia in humans is associated with RET mutations. *American Journal of Human Genetics*.

[52] Clarke JC, Patel SR, Raymond RM (2006). Regulation of c-Ret in the developing kidney is responsive to Pax2 gene dosage. *Human Molecular Genetics*.

[53] Rosselot C, Spraggon L, Chia I (2010). Non-cell-autonomous retinoid signaling is crucial for renal development. *Development*.

[54] Takahashi M (2001). The GDNF/RET signaling pathway and human diseases. *Cytokine and Growth Factor Reviews*.

[55] Jain S (2009). The many faces of RET dysfunction in kidney. *Organogenesis*.

[56] Airaksinen MS, Saarma M (2002). The GDNF family: signalling, biological functions and therapeutic value. *Nature Reviews Neuroscience*.

[57] Jain S, Encinas M, Johnson EM, Milbrandt J (2006). Critical and distinct roles for key RET tyrosine docking sites in renal development. *Genes and Development*.

[58] Jain S, Knoten A, Hoshi M (2010). Organotypic specificity of key RET adaptor-docking sites in the pathogenesis of neurocristopathies and renal malformations in mice. *Journal of Clinical Investigation*.

[59] Pepicelli CV, Kispert A, Rowitch DH, McMahon AP (1997). Rapid communication: GDNF induces branching and increased cell proliferation in the ureter of the mouse. *Developmental Biology*.

[60] Michael L, Davies JA (2004). Pattern and regulation of cell proliferation during murine ureteric bud development. *Journal of Anatomy*.

[61] Meyer TN, Schwesinger C, Bush KT (2004). Spatiotemporal regulation of morphogenetic molecules during in vitro branching of the isolated ureteric bud: toward a model of branching through budding in the developing kidney. *Developmental Biology*.

[62] Kuure S, Cebrian C, Machingo Q (2010). Actin depolymerizing factors cofilin1 and destrin are required for ureteric bud branching morphogenesis. *PLoS Genetics*.

[63] Kuure S, Chi X, Lu B, Costantini F (2010). The transcription factors Etv4 and Etv5 mediate formation of the ureteric bud tip domain during kidney development. *Development*.

[64] Powers CJ, McLeskey SW, Wellstein A (2000). Fibroblast growth factors, their receptors and signaling. *Endocrine-Related Cancer*.

[65] Arman E, Haffner-Krausz R, Chen Y, Heath JK, Lonai P (1998). Targeted disruption of fibroblast growth factor (FGF) receptor 2 suggests a role for FGF signaling in pregastrulation mammalian development. *Proceedings of the National Academy of Sciences of the United States of America*.

[66] Deng CX, Wynshaw-Boris A, Shen MM, Daugherty C, Ornitz DM, Leder P (1994). Murine FGFR-1 is required for early postimplantation growth and axial organization. *Genes and Development*.

[67] Qiao J, Bush KT, Steer DL (2001). Multiple fibroblast growth factors support growth of the ureteric bud but have different effects on branching morphogenesis. *Mechanisms of Development*.

[68] Ohuchi H, Hori Y, Yamasaki M (2000). FGF10 acts as a major ligand for FGF receptor 2 IIIb in mouse multi-organ development. *Biochemical and Biophysical Research Communications*.

[69] Sims-Lucas S, Cullen-McEwen L, Eswarakumar VP (2009). Deletion of Frs2*α* from the ureteric epithelium causes renal hypoplasia. *American Journal of Physiology*.

[70] Passos-Bueno MR, Wilcox WR, Jabs EW, Sertié AL, Alonso LG, Kitoh H (1999). Clinical spectrum of fibroblast growth factor receptor mutations. *Human Mutation*.

[71] Maeshima A, Vaughn DA, Choi Y, Nigam SK (2006). Activin A is an endogenous inhibitor of ureteric bud outgrowth from the Wolffian duct. *Developmental Biology*.

[72] Michos O, Cebrian C, Hyink D (2010). Kidney development in the absence of Gdnf and Spry1 requires Fgf10. *PLoS Genetics*.

[73] Eswarakumar VP, Lax I, Schlessinger J (2005). Cellular signaling by fibroblast growth factor receptors. *Cytokine and Growth Factor Reviews*.

[74] Lu BC, Cebrian C, Chi X (2009). Etv4 and Etv5 are required downstream of GDNF and Ret for kidney branching morphogenesis. *Nature Genetics*.

[75] Holbro T, Hynes NE (2004). ErbB receptors: directing key signaling networks throughout life. *Annual Review of Pharmacology and Toxicology*.

[76] Melenhorst WBWH, Mulder GM, Xi QI (2008). Epidermal growth factor receptor signaling in the kidney key roles in physiology and disease. *Hypertension*.

[77] Bernardini N, Bianchi F, Lupetti M, Dolfi A (1996). Immunohistochemical localization of the epidermal growth factor, transforming growth factor *α*, and their receptor in the human mesonephros and metanephros. *Developmental Dynamics*.

[78] Threadgill DW, Dlugosz AA, Hansen LA (1995). Targeted disruption of mouse EGF receptor: effect of genetic background on mutant phenotype. *Science*.

[79] Woolf AS, Kolatsi-Joannou AS, Hardman P (1995). Roles of hepatocyte growth factor/scatter factor and the met receptor in the early development of the metanephros. *Journal of Cell Biology*.

[80] Schmidt C, Bladt F, Goedecke S (1995). Scatter factor/hepatocyte growth factor is essential for liver development. *Nature*.

[81] Liu Y, Chattopadhyay N, Qin S (2009). Coordinate integrin and c-Met signaling regulate Wnt gene expression during epithelial morphogenesis. *Development*.

[82] Ishibe S, Joly D, Zhu X, Cantley LG (2003). Phosphorylation-dependent paxillin-ERK association mediates hepatocyte growth factor-stimulated epithelial morphogenesis. *Molecular Cell*.

[83] Ishibe S, Haydu JE, Togawa A, Marlier A, Cantley LG (2006). Cell confluence regulates hepatocyte growth factor-stimulated cell morphogenesis in a *β*-catenin-dependent manner. *Molecular and Cellular Biology*.

[84] Karihaloo A, Nickel C, Cantley LG (2005). Signals which build a tubule. *Nephron Experimental Nephrology*.

[85] O’Brien LE, Tang K, Kats ES, Schutz-Geschwender A, Lipschutz JH, Mostov KE (2004). ERK and MMPs sequentially regulate distinct stages of epithelial tubule development. *Developmental Cell*.

[86] Ferrara N (2004). Vascular endothelial growth factor: basic science and clinical progress. *Endocrine Reviews*.

[87] Kanellis J, Mudge SJ, Fraser S, Katerelos M, Power DA (2000). Redistribution of cytoplasmic VEGF to the basolateral aspect of renal tubular cells in ischemia-reperfusion injury. *Kidney International*.

[88] Lindahl P, Hellström M, Kalén M (1998). Paracrine PDGF-B/PDGF-R*β* signaling controls mesangial cell development in kidney glomeruli. *Development*.

[89] Heldin CH, Östman A, Rönnstrand L (1998). Signal transduction via platelet-derived growth factor receptors. *Biochimica et Biophysica Acta*.

[90] Eitner F, Ostendorf T, Kretzler M (2003). PDGF-C expression in the developing and normal adult human kidney and in glomerular diseases. *Journal of the American Society of Nephrology*.

[91] Nakagawa T, Izumino K, Ishii Y (2011). Roles of PDGF receptor-beta in the structure and function of postnatal kidney glomerulus. *Nephrology Dialysis Transplantation*.

[92] Hammerman MR, Rogers S (1987). Distribution of IGF receptors in the plasma membrane of proximal tubular cells. *American Journal of Physiology*.

[93] Duong Van Huyen JP, Amri K, Bélair MF (2003). Spatiotemporal distribution of insulin-like growth factor receptors during nephrogenesis in fetuses from normal and diabetic rats. *Cell and Tissue Research*.

[94] Rogers SA, Ryan G, Hammerman MR (1991). Insulin-like growth factors I and II are produced in the metanephros and are required for growth and development in vitro. *Journal of Cell Biology*.

[95] Bueno MP, Guadagnini D, Gonçalves FLL (2010). Assessment of the expression of IR*β*, IRS-1, IRS-2 and IGF-IR*β* in a rat model of intrauterine growth restriction. *Fetal Diagnosis and Therapy*.

[96] Yosypiv IV (2009). Renin-angiotensin system-growth factor cross-talk: a novel mechanism for ureteric bud morphogenesis. *Pediatric Nephrology*.

[97] Oshima K, Miyazaki Y, Brock JW, Adams MC, Ichikawa I, Pope JC (2001). Angiotensin type II receptor expression and ureteral budding. *Journal of Urology*.

[98] Lacoste M, Cai Y, Guicharnaud L (2006). Renal tubular dysgenesis, a not uncommon autosomal recessive disorder leading to oligohydramnios: role of the renin-angiotensin system. *Journal of the American Society of Nephrology*.

[99] Besset V, Scott RP, Ibáñez CF (2000). Signaling complexes and protein-protein interactions involved in the activation of the Ras and phosphatidylinositol 3-kinase pathways by the c-Ret receptor tyrosine kinase. *Journal of Biological Chemistry*.

